# Anxiety and panic fear in adults with asthma: prevalence in primary care

**DOI:** 10.1186/1471-2296-8-62

**Published:** 2007-10-26

**Authors:** Cindy L Cooper, Glenys D Parry, Carol Saul, Alyn H Morice, Bruce J Hutchcroft, Julia Moore, Lisa Esmonde

**Affiliations:** 1 ScHARR, University of Sheffield, Sheffield, UK; 2 Academic Department of Medicine, University of Hull, Cottingham, UK; 3 Department of Respiratory Medicine, Sheffield Teaching Hospitals Trust, Sheffield, UK

## Abstract

**Background:**

Patients may find it difficult to distinguish between the symptoms of anxiety and those of asthma. Findings are equivocal on whether there is a specific link between anxiety and asthma. The aims of this study were to i) to identify the prevalence of anxiety, depression and panic fear in adults with asthma compared with that of the general population ii) to investigate whether there is a specific relationship between asthma and anxiety.

**Methods:**

An epidemiological survey of 872 adults with a diagnosis of asthma identified from six General Practices in Sheffield, England. Community postal survey using self-completion questionnaire.

**Results:**

The response rate was 59%. People with asthma had higher mean Hospital Anxiety and Depression Scale (HADS) anxiety scores than UK norms with a higher proportion above the clinical cut-off. Mean HADS depression scores were significantly higher than UK norms and norms for a general population sample of people registered with the same practice. These effects were age-related with the relationship between asthma and psychological distress most marked over the age of 45. The prevalence of asthma-specific panic fear was 15.7%.

**Conclusion:**

A significant minority of people have high levels of panic fear (as measured by the Asthma Symptom Checklist) associated with asthma. However, in adults with asthma there is also high prevalence of both generalised anxiety and depression (as measured by the HADS), suggesting that the link of anxiety to asthma may be part of a broader relationship between psychological distress and chronic disease rather than a specific one.

## Background

There has been considerable research on the relationship between asthma and anxiety. Recent reviews [1-3] of the literature describe an increased rate of psychological co-morbidity in adults with asthma including anxiety and panic symptoms. Although anxiety is a normal reaction to extreme dyspnea [4], and may even be functional in moderation [5,6], high levels of panic fear may lead both to exacerbation of the condition and poor management. For example, it has been demonstrated that panic may trigger an asthma attack through hyperventilation and airways cooling [7]. Patients may find it difficult to distinguish between the symptoms of anxiety and those of asthma leading to inappropriate self-medication [8]. A New Zealand study of management errors in acute severe asthma in adults found that most were related to patient behaviour, and that panic and the overuse of beta-agonists were important contributory causes [9]. In a study of the Asthma Symptom Checklist [10] used in people with asthma recruited from general practice, the responses to the panic fear subscale predicted oral steroid intake and the authors concluded that the emotional impact of asthma showed a stronger association with aspects of asthma management than symptoms of dyspnea did. Similarly, anxiety has been shown to be related levels of oral corticosteroid prescribing in near-fatal asthma patients [11].

Findings are equivocal on whether there is a specific link between panic and respiratory disorders including asthma. The high prevalence of panic-fear or anxiety symptoms in asthma populations does not in itself establish a specific link as this could be part of a broader relationship between any chronic disease and lowered psychological wellbeing. A Swedish research group found significant correlation of anxiety and depression with self-reported respiratory symptoms, but not objective asthma variables using the Hospital Anxiety and Depression Scale [12]. This finding was replicated in asthma patients treated in GP practices in the UK, where there were significant correlations of HADS anxiety and depression with self-reported symptoms from the Asthma Quality of Life Questionnaire, but not with objective measures of lung function [13]. A recent review [14] of the literature reported that the findings are inconclusive on whether people with asthma are more likely to be depressed than those without asthma. Although HADS anxiety has been estimated in community samples of adults with asthma, ASC panic fear, a measure of asthma specific anxiety, has previously been less frequently reported in community populations. The higher rates of panic in hospital attendees could be a selection bias of hospital attendance, in that these rates may be associated with attendance at hospital. This paper addresses the question of whether the higher levels of anxiety demonstrated in people with asthma is specifically linked or whether it is part of a picture of reduced psychological well-being by exploring the prevalence, in adults with asthma, of anxiety and panic fear as well as the prevalence of depression which has not previously been conclusively established.

### Research questions

1. What is the prevalence of anxiety, panic fear and depression in adults with asthma, irrespective of their consultation history?

2. Is this prevalence significantly higher than that found in a general population survey of health and illness in the same General Practices and with UK normative data?

3. Are anxiety scores rather than depression scores elevated, compared with the general population, to support the suggestion of a specific relationship between asthma and anxiety?

4. Does the comparison of anxiety and panic fear levels with those in clinic-based samples suggest a selection bias of hospital attendance in previous research?

Throughout this paper levels of anxiety, panic fear and depression are reported as assessed using generally agreed cut-off points on established measures (HADS and ASC) and not as clinically diagnosed psychological disorders.

## Methods

A random sample of 872 adults with asthma was obtained from six General Practices (serving a population of 32,343 listed permanent adult patients) in Sheffield, England during 1998 and 1999. Practices were selected to represent a wide range of Townsend deprivation scores (from 4.9 to -2.6). The six practices had a total of 2169 adult patients between the ages of 18 and 65 with a diagnosis of asthma registered with them. A random sample of 872 (approximately 40% of registered adults with asthma) was identified using computer generated random numbers. Each patient received a letter from the GP inviting him or her to complete and return an enclosed questionnaire.

The questionnaire included demographic questions, and a screening tool comprising the Asthma Symptom Checklist (ASC) and the Hospital Anxiety and Depression Scale (HADS).

The ASC [15] is a self-report questionnaire widely used in asthma research to assess subjective asthma symptomatology including panic-fear. It was derived from empirical cluster analysis in a hospital sample, replicated in a later study [15,16] and has been validated in the UK [17]. We used a nine item version of ASC panic fear with response options of 1 to 5 and a cut off score of 28 and above. The cut off was derived from conventional practice of considering an average item score of 3 or more to be in the clinical range [16]. A more recent reevaluation of the ASC demonstrated that, although some of the items included in the panic fear scale may be redundant, the original five factors, including one representing panic-fear, were replicated and the internal consistency of the panic fear scale was good [10].

The HADS is a brief (14 item) scale developed as a reliable, practical and valid tool for identifying and quantifying anxiety and depression in patients in hospital clinics [18]. It has been extensively used in hospital and primary care patients and in the general population and reviews of its use confirm its value as a case finder for anxiety disorders and depression in these populations [19,20]. The internal consistency of the HADS has been well demonstrated and optimal balance between sensitivity and specificity for HADS as a screening tool is achieved using a cut-off of 8+ for both HAD anxiety and depression subscales [20].

Strategies for increasing the response rate to postal questionnaires were employed [21] including sending two reminders to non-respondents.

Ethical approval for this study was obtained from both North and South Sheffield Local Research Ethics Committees.

### Comparison data

HADS anxiety and depression scores were compared with Crawford's community sample of 1792 UK adults [22]. In Crawfords study HADS data were collected from 1792 members of the general adult population (females = 978, males = 810). Participants were recruited from a wide variety of sources including commercial and public service organizations, community centres and recreational clubs. The mean age of the sample was 41.5 years (SD = 15.9, range = 18–91). Each potential participant received an introductory letter, a HADS form and a form for recording demographic variables. The refusal rate was approximately 18%.

HADS depression scores were also compared with those of patients from the same General Practices, a subset of the Sheffield Health and Illness Survey (SHAIPS) [23]. SHAIPS was a postal survey of a representative sample of the population of Sheffield looking at health status and use of health services and included the HADS depression scale. It was carried out two years after the survey reported here. The sampling frame used was the local population health register which contains, amongst other data, the GP with whom the person is registered. Stratification was used to ensure that the sample was representative of the Sheffield population in terms of age, sex and electoral ward. It is likely that some individuals responded to both surveys but the number doing so is unknown.

### Data analysis

Data analysis was undertaken using SPSS software (version 10). The chi^2 ^test was used to compare proportions of clinically significant scores for HADS and ASC measures between men and women, chi^2 ^for trend for differences by age group, and Spearman's rank correlation was used to determine any relationship between continuous age and HADS/ASC score. Analysis of variance was used to compare HADS anxiety and depression scores with data from the UK and the general population of Sheffield. t-tests were used for comparison of means. Ideally, multiple regression analysis would have been used to determine whether there were differences in HADS depression by age and sex between the study population and the general population of Sheffield. However, as we did not have access to the SHAIPs raw data we undertook individual t-tests on each age, sex combination.

### Data oversight

The investigators who collected and analysed the data reported to, and were advised by, a data monitoring group independent of the researchers.

## Results

### Respondents

461 completed questionnaires were received giving a crude response rate of 53%. After accounting for 89 questionnaires returned either undelivered or marked indicating that the recipient did not have asthma the adjusted response rate was 59%.

We had a higher proportion of responses from women – 38.3% of respondents were male and 61.7% female. This approximately reflects the sex distribution of the Sheffield population and a higher prevalence of asthma in women in Sheffield [23].

The mean age of respondents was 42.4 (S.D = 13.4) years, the median was 41 years and the range was 18–66 years. The % of respondents in each age group approximately reflects the age distribution of the Sheffield population except for a slightly lower response rate in the younger age group [24].

### HADS anxiety

The proportion of patients with a HADS anxiety score of 10 or more was 31.6%. HADS anxiety score increased with age for both scores 8+ (chi^2 ^= 10.602, p = 0.031) and 10+ (chi^2 ^= 21.218, p < 0.001). There was no significant difference between men and women with respect to these proportions. However, there was a significant difference in mean HADS anxiety scores by sex (Mann-Whitney U = -2.916, p = 0.004). Women had a mean score of 8.2 (S.D = 4.5) compared with a mean score of 6.9 (S.D. = 4.3) for men. The proportion of people with asthma with anxiety at a level of possible clinical disorder (HADS anxiety score 8–10) is no different from the general population. However, the proportion with probable clinical disorder (scoring 11+), is double that in the general population. The cut off of 11+ is used here for direct comparison with UK normative data^22^. Mean HADS anxiety scores were also significantly higher for the asthma sample, compared with UK norms (Table 1).

**Table 1:** Hospital Anxiety and Depression Scale anxiety: Comparison of asthma population survey data with population norms [22]

	Asthma Population	Population norm (Crawford et al)	Difference	95% CI	p-value
n	455	1792			
Median	7.0	6.0			
Mean (sd)	7.79 (4.45)	6.14 (3.76)	+1.65	+1.23 to +2.05	<0.001
% score 8+	47.3%	33.2%	+0.14.1%	+9.0 to +19.1%	
% score 8–10	20.9%	20.6%	+0.3%	3.7 to +4.7%	
% score 11–15	20.2%	10.0%	+10.2%	+6.5 to +14.4%	
% score 16+	5.9%	2.6%	+3.3%	+1.3 to +6.0%	

### HADS depression

The proportion of patients with a HADS depression score of 10 or more was 13.6%. HADS depression score increased with age for both scores 8+ (chi^2 ^= 47.988, p < 0.001) and 10+ (chi^2 ^= 30.721, p < 0.001). There were no significant differences between men and women.

Mean HADS depression scores were significantly higher for the asthma sample, compared with UK norms (Table 2). The proportion of the asthma population with depression at levels of both possible and probable clinical disorder was significantly greater than UK normative data (Table 2).

**Table 2:** Hospital Anxiety and Depression Scale depression: Comparison of asthma population survey data with population norms [22]

	**Asthma population**	**Population norm (Crawford et al)**	**Difference**	**95% CI**	**p-value**
n	454	1792			
Median	4.0	3.0			
Mean (sd)	4.72 (4.08)	3.68 (3.07)	+1.04	+0.67 to +1.42	<0.001
% score 8+	22.3%	11.4%	+10.9%	+7.0 to +15.1%	
% score 8–10	13.0%	7.8%	+5.2%	+2.1 to +8.8%	
% score 11–15	7.9%	2.9%	5.0%	+2.7 to +8.0%	
% score 16+	1.3%	0.7%	+0.6%	-0.3 to 2.2%	

Mean HADS depression scores were also significantly higher for the respondents of our survey compared with Sheffield general population data, for the total sample, and for men and women separately (Table 3). This effect was accounted for by the older age groups (45–54 years and 55–64 years): see Table 3.

**Table 3:** Mean Hospital Anxiety and Depression Scale depression score: Comparison of asthma population survey data with Sheffield Health and Illness Prevalence Survey (SHAIPS) findings for the same six practices

		**Total**	**Males**	**Females**
All ages	Asthma	4.70	4.64	4.72
	SHAIPS (6 practices)	3.58	3.62	3.55
	p-value	<0.001	<0.01	<0.001
18–34	Asthma	3.34	3.33	3.36
	SHAIPS (6 practices)	3.02	2.73	3.23
	p-value	= 0.298	= 0.212	= 0.763
35–44	Asthma	3.39	2.92	3.73
	SHAIPS (6 practices)	3.45	3.38	3.51
	p-value	= 0.882	= 0.498	= 0.708
45–54	Asthma	6.24	6.66	6.02
	SHAIPS (6 practices)	4.18	4.60	3.85
	p-value	<0.001	= 0.025	<0.001
55–64	Asthma	6.44	6.97	6.12
	SHAIPS (6 practices)	4.21	4.48	3.97
	p-value	<0.001	<0.001	<0.01

The proportion of the asthma population sample with high HADS depression scores was significantly higher for all categories:- 8+ and 11+ (Table 4).

**Table 4:** Prevalence of Hospital Anxiety and Depression Scale (HADS) depression: Comparison of asthma population survey data with Sheffield population norms. Standardised for age and sex

Study	Standardised prevalence (95% CI) of HAD depression:	
	
	8+	11+
Asthma (n = 441)	24.3% (20.5 to 28.5%)	9.2% (6.9 to 12.4%)
SHAIPS: Sheffield normative (n = 714)	12.2% (10.0 to 14.8%)	5.4% (4.0 to 7.4%)

### Asthma specific panic fear

The prevalence of asthma-specific anxiety from the ASC Panic Fear score was 15.7% (62/395). Women were significantly more likely than men to experience panic fear associated with asthma: 19.2% (46/239) compared with 10.6% (16/151, Mann-Whitney U = -2.498, p < 0.05). The mean panic-fear ASC score (S.D.) was 17.9 (8.5) overall and 16.7 (8.3) for men compared with 18.7 (8.7) for women. The range was 9 – 44. The mean score for women was significantly higher than that for men (Mann-Whitney U = -2.498, p = 0.012). There was a significant correlation between increase in ASC score and increased in age (r = 0.114, p < 0.05).

## Discussion and conclusion

### Summary of main findings

The results of this study clearly show that both the prevalence of anxiety and the level of anxiety experienced by people with asthma (as measured using the HADS) are higher than that of the general population. Almost one third of asthma sufferers experience anxiety at the level of probable clinical disorder, double that in the general population. The prevalence of anxiety is higher in women than in men with asthma and increases with age. A significant minority of people with asthma experience asthma specific anxiety as measured by the panic fear subscale of the Asthma Symptom Checklist. However, the fact that the prevalence of generalised anxiety and depression are high in this population indicates that anxiety experienced in asthma may be part of a broader picture of generalised psychological distress rather than indicating that panic has a specific role in asthma.

### Comparison with existing literature

These results from a general population sample are important in that they support the evidence of panic reported previously for hospital attenders [11,25], demonstrating that the findings are not only seen in hospital attenders but generally for people with asthma. Our findings are consistent with those reported previously. Rimington [13] found that 30% of patients with a diagnosis of asthma recruited through general practice had anxiety scores of 11 or more on the HADS suggestive of a clinical anxiety state, for inner city patients this rate was 40%. In the same study 10% of patients had HADS scores suggesting depression with mean scores (S.D) of 3.7 (2.7) and 6.0 (4.3) for suburban and inner city patients respectively compared with our findings of a mean HADS depression score of 4.72 (4.08). An Australian study [26] reported a prevalence of anxiety (scoring 8 or more on HADS) of 40% of patients with asthma. More-over the findings add weight to the evidence supporting increased levels of depression in asthma [14].

The results also corroborate the general findings of high levels of panic experienced by people with asthma reported in a recent review [1]. Specific comparison of our results with other published studies is not straightforward as variations of the ASC panic fear subscale are used. The ASC mean scores reported in this paper are some-what higher than those reported for a comparable study [10] of asthma patients recruited from General Practice but the significantly higher levels in women compared with men is replicated.

That higher levels of anxiety and depression are associated with increasing age in the asthma population is interesting. In other chronic conditions psychological distress has been shown to decrease with age [27], as it does in the general population [28]. The present findings may be explained by evidence that the level of depression experienced in asthma is related to the level of illness uncertainty [29]. It may be that older people may feel more uncertain about managing their asthma. An alternative explanation may be that psychological distress increases with increasing duration of asthma as reported previously in men [30]. However, the upper age limit in this study was 65 years so the older age group were somewhat younger than comparable studies [27].

### Strengths and limitations of this study

In the present study, duration of illness was not recorded so that it is not possible to distinguish between association of asthma with age or duration of illness. It is also likely that a proportion of participants may have had Chronic Obstructive Pulmonary Disease which occurs from around 45+ years and has been shown to have a major impact on QOL and mood [31]. This may explain the link between depression and age. In this study the GP diagnosis of asthma was not verified though the upper age limit was restricted to 65 as the likelihood of COPD increases significantly over 65 years.

A limitation of the study is that the sample was drawn from one UK city. Comparisons were made with data from the general population from the same GP practices indicating that the findings of psychological distress are related to the experience of asthma or chronic disease rather than being related to the specific location. However, a recent, comparable, community study reported a prevalence of 'possible' anxiety of 38% and 'probable' anxiety of 18.9% in people with asthma and prevalence levels of depression were no greater than controls [32]. This was carried out in a relatively affluent rural, mainly Caucasian population, indicating that prevalence of psychological distress associated with asthma may vary with geographical and socioeconomic factors. An earlier study reported significant differences in the level of anxiety and depression experienced by people with asthma living in a suburban compared with an inner city location [13].

A further limitation is that no attempt was made to assess the prevalence of clinically diagnosed anxiety, depression or panic fear in the population.

A strength of this study is the relatively large sample size, however it is acknowledged that lack of information related to possible non-response bias is a weakness. Care was also taken to ensure that the socio-economic demographics of the practice populations reflected that of the city as a whole.

### Implications for future research and clinical practice

In our parallel study [33] into the management of anxiety in asthma, GPs had difficulties identifying clinically anxious patients. This indicates that anxiety related complications of asthma might not be recognised in general practice despite high prevalence levels. The screening tool used in this study could be used in both general practice and hospital clinics to identify patients with asthma experiencing levels of anxiety that may affect their ability to manage their asthma. Once such patients are identified, providing effective interventions to meet their psychological needs may pose a challenge to community services. Further research is needed into effectiveness of interventions to help people with asthma manage the associated psychological distress, particularly interventions which can be delivered by those staff already in contact with patients such as the asthma clinic nurse.

## Competing interests

The author(s) declare that they have no competing interests.

## Authors' contributions

CLC was the study manager responsible for undertaking all aspects of the study and for drafting this paper. GDP was the principal investigator with responsibility for the conception, design and conduct of the study and was also involved in drafting the paper. CS undertook data analysis and drafting for the paper. AHM was involved in the conception of the study. Both AHM and BJH advised on the implementation of the study, interpretation of the data and were involved in revising the article critically for intellectual content. JM and LE undertook the data collection. All authors have approved the final version of this paper.

## Pre-publication history

The pre-publication history for this paper can be accessed here:


